# Supplementary material for: Prevention and Control of COVID-19 in Imperfect Condition: Practical Guidelines for Nursing Homes by Japan Environment and Health Safety Organization (JEHSO)
Source: Int J Environ Res Public Health. 2021 Sep 28;18(19):10188. doi: 10.3390/ijerph181910188 (PMC8508057; doi:10.3390/ijerph181910188)
Supplement: Supplementary file 1 [file ijerph-18-10188-s001.zip › ijerph-1362018-supplementary.pdf]

Supplementary Table

Table S1. Checklists for daily practice.

**I. Items common in any service businesses including restaurants, nursing homes, schools, libraries**
**1. Thorough hand washing and disinfection and use of mask**
□ Service users and employees are fully informed of their need for mask use.
□ Masks are distributed to users who are not wearing them so that people without specific conditions can wear it.
□ Disinfecting equipment is installed in various places. *The ingredients must be effective against SARS-CoV-2.
□ Service users are fully informed of the need for thorough hand washing and disinfection through posted notices and other methods.
□ Employees are fully informed of the need for thorough hand washing and disinfection through posted notices and other methods.
□ The use of hand dryers and shared towels are prohibited and paper towels are installed.
□ Staff areas, such as employee breakrooms, are frequently disinfected.
□ Other hygiene management is thoroughly performed. (Specify)
**2. Droplet avoidance and distance for infection prevention (maintain distance as much as possible, rough indication 2m)**
□ Crowds are avoided by management of lines, placement of floor markings, and use of online booking of appointments.
□ Distance between individuals, including employees, is maintained by methods such as seating arrangements.
□ Individuals are made aware of the need to avoid talking loudly.
□ The risk of contact is reduced by installation of screens in areas where face-to-face meetings are expected.
**3. Avoidance of the "3 C's – closed spaces with poor ventilation, crowded places, and close-contact settings."**
□ If situations where the 3 C's or loud conversations are expected, numbered tickets for seating are issued, or the number of people and/or length of time is limited.
□ Rooms are routinely ventilated by the opening of doors and windows.
□ Rooms, such as employee breakrooms, are ventilated as much as possible.
□ Employees are to avoid eating meals face-to-face or conversing face-to-face.
**4. Cleaning and disinfection of facility**
□ The number of areas and items touched by multiple people are reduced as much as possible. Problematic areas are cleaned/disinfected frequently.
□ Gloves and masks are worn during cleaning, disinfecting, or garbage collection.
□ Hands are thoroughly washed/disinfected after cleaning, disinfecting, or garbage collection.
**5. Health management of service users and employees**
□ Employees thoroughly wash/disinfect their hands after completion of each work process.
□ Employees make efforts as much as possible so that service users with fever not to enter the facility.
□ Employees measure their temperature and check their health condition before going to work and record/report them daily.
□ Preparations are made in case a patient is infected, such as keeping track of number of visitors and verifying the public health center under whose jurisdiction the facility operates.
□ If employees do not feel well, they are encouraged to recuperate at home. If employees become ill at work, they are encouraged to return home.
□ The facility enforces the practice of not touching the face with gloved hands.

□ Dirty gloves are frequently replaced with new ones.
**II. Items to check specifically for nursing homes**
Staff education
□ A person is assigned to be in charge of infection control.
□ An affiliated medical institution is chosen in case problems regarding infection control cannot be resolved within the facility.
□ A manual on infection prevention is created and employees are fully informed of it.
□ A study group for infection control is created for employees and meets regularly.
□ Employees are fully informed of proper hand washing methods.
□ After completing each task, employees thoroughly wash their hands using running water (and soap) or thoroughly disinfect their hands using alcohol.
□ Employees wash their hands under running water before touching other items or areas, if their hands are visibly dirty such as from body fluids (including blood and urine) or feces.
□ Employees are to dry their hands thoroughly before beginning their next task.
Installation of disinfectants
□ A facility for hand washing or disinfecting is installed at regular intervals.
□ Liquid soap is used for hand washing and dispensers are not refilled by pouring additional soap.*
□ For hand washing, employees are instructed to turn on and off a faucet without directly touching the faucet handle with their hands (instead using the elbow or a paper towel).
□ Paper towels are installed so that they can be used properly without contamination.
Prevention of infection through contact
□ When employees are to come in contact with body fluids (including blood and urine) or feces, they wear gloves. Then they change the gloves and disinfect their hands after each incident.
□ Employees dispose of their used gloves after sealing them in a bag, avoiding any contact with other items.
□ Employees always wash or disinfect their hands after removing the gloves.
□ Employees wear gloves and plastic gowns or aprons if droplets are expected to occur such as from body fluids (including blood and urine) or feces.
□ Employees wear eye protection when giving a bed bath or oral cleaning with swab/gauze and disinfect it each time after completion of the task.
□ Plastic gowns and gloves are changed into new ones after each patient.
□ Used gowns and aprons are sealed in bags and disposed of, avoiding any contact with other items.
□ Employees are fully informed that they should avoid wearing their plastic aprons used for bed bathing patients when they walk in hallways or at nurse stations.
□ Clean items and used items are transported in separate carts, or used items are stored in the lower part of the same cart. For example, used towels and used diapers are not placed at the same level of a cart as new ones when bed bathing patients; and used tableware after a meal and clean tableware before serving are not placed on the same cart.
□ Floors of living rooms are cleaned daily using a solution containing a surfactant and using a tool, such as a mop (wet method).
□ Employees use a mop with a disposable cleaning sheet or a mop that has been washed and dried thoroughly.
□ Cleanliness of bathrooms (toilet bowls) is maintained.
□ Dirty clothes and linen are placed in a bag (such as a laundry bag or plastic bag) at the point of use, transported, and cleaned.
□ Contaminated clothes and linen (with a risk of spreading infection) are washed with detergent or bleach, or are changed to brand new ones.

□ Clean linen is stored in a linen cabinet.
□ Only clean items are placed in a linen cabinet.
□ Sinks for clean and dirty items are kept separate to avoid mixing clean and dirty items around the sink areas.
Mindset of employees
□ Employees always wear masks when interacting with patients.
□ Employees are to avoid going to work if they have a cough or fever.
□ Lab coats and uniforms are regularly changed to clean ones. If they become dirty with body fluids (such as blood or sputum), they are changed to clean ones at that time.
□ Employees clearly understand that they should refrain from touching their face or hair while on duty.
Patient care
□ If a patient develops a fever, employees take measures such as isolating him/her in a private room.
□ If a service user is infected, then he/she is to avoid contact with other users as much as possible.
□ Employees are made aware of infected service users using tools such as a notice board.
□ Transporting of individuals with fever is restricted as much as possible.
□ If a service user has a continuing cough or sputum production, employees make efforts in early SARS-Cov-2 detection, such as by having a user seek care at a medical facility.
□ If a service user has a risk to transmit the infection via droplets and cannot be isolated from other users, employees create sufficient space between them, such as by room dividers – including partitions – or by placing beds at least 2 m apart.
□ Transporting is restricted as much as possible for a service user who has a risk to transmit the infection via droplets, and the user is asked to wear a mask, as necessary.
□ Employees clean with disinfecting alcohol, areas which are routinely touched by a service user who has a risk to transmit infection via contact (including bed rails, tables, door knobs, and handrails)
□ Employees use detergent or bleach to thoroughly wash or disinfect tableware used by a service user with a risk to transmit the infection via contact.
□ Employees use disinfectant, such as disinfecting alcohol, to clean devices (including thermometers, stethoscopes, and blood pressure monitors) used on a service user with a risk to transmit the infection via contact. Then the employees remove the devices out of the room.
□ Service users are instructed on proper hand washing methods and encouraged to practice diligent hand washing.
□ Service users are instructed to use personal towels or paper towels in places such as washrooms and toilets, and to avoid sharing cloth towels.
□ Service users who have a cough are asked to wear masks, if they do not weare it due to dementia etc.
□ Employees ensure to prohibit the sharing of personal hyginene items e.g. razors, toothbrushes, towels by each patient.
□ Service users are given opportunities to be vaccinated against diseases such as influenza.
□ A system is used in which employees are assigned to be in charge of care for infected service users.

* Refilling by pouring additional soap may cause contamination with bacteria that can proliferate in soap, such as p*seudomonas aeruginosa*.
